# Ecotoxicity, Health Risks and Contact Allergy Due to p-Phenylenediamine in Hair Dyes and Tattoos: Female Students' Perspectives

**DOI:** 10.7759/cureus.60984

**Published:** 2024-05-24

**Authors:** Shabihul Fatma Sayed, Hamad G Dalai, Mukti Sharma, Raneem Halawani

**Affiliations:** 1 Nursing, College of Nursing, Jazan University, Jazan, SAU; 2 Research, Private Practice, Aligarh, IND; 3 Nursing, Farasan University College, Jazan University, Farasan Island, SAU

**Keywords:** oxidative radicals, contact allergy, ppd, health risks, ecotoxicity

## Abstract

While the financial advantages of hair coloring and tattooing are widely acknowledged, environmental hazards and health risks linked to this trend due to their p-phenylenediamine (PPD) content have received less attention. Health education on hair-dying products is warranted to enhance the public's awareness of hair-dying ingredients and their side effects.

A cross-sectional study was therefore conducted with 319 students to assess knowledge of ecotoxicity, health risks, and practices of hair dyeing and tattooing among undergraduate students. A random sample of 59 students was checked for any allergic morphology in the scalp and exposed areas of skin near the neck, ears, palms, and nails. Responses collected were used for data analyses using IBM SPSS Statistics for Windows, Version 17 (Released 2008; IBM Corp., Armonk, New York, United States).

Use of hair dye was significantly high among study participants 58.5% (n=187; p<0.05). However, their knowledge regarding the presence of PPD in hair dyes and associated environmental toxicity (37.8%, n=121) was very limited. The majority of participants did not do any allergy tests before applying hair dye (88.9%, n=283). The study revealed that the main reason for hair coloring was as a fashion statement (93.7%, n=299). Regarding tattooing practices, 96.9% (n=309) of study participants had never practiced tattoos, and hence, the prevalence of tattooing was 3.9% (n=12).

These data confirmed that the practice of hair dyeing as a style statement was high among students. However, the majority were unaware of their PPD contents and their potential ecotoxicity and health risks.

## Introduction

It is innate in human nature to want to look better. Hair dyes (HDs), one of the first cosmetics, have been used for centuries and in many cultures. Because lifestyles are changing so quickly, these products are vital and contribute positively to our standard of living. According to SCCP, 9th Forum Brussels, Belgium, p-phenylenediamine is an organic molecule having the molecular formula C6H8N2 [1]. It is the primary ingredient in the HD that causes toxicity. To intensify its coloring effects, it is mixed with H_2_O_2_ which is extremely soluble. 

Considering the degree and frequency of human contact with hair coloring products, their ingredients must be safe. While the socio-economic benefits and impacts of this globalization trend are widely understood, the environmental effects and adverse health impacts are largely unknown. In particular, commonly available oxidative dyes potentially pose specific environmental risks due to the aromatic amine p-phenylenediamine (PPD).

According to a 2019 global market research report, the hair coloring industry is predicted to reach USD 40.08 billion by 2024. PPD forms a variety of hues by penetrating the hair shaft and binding to proteins [1-3]. HD wastewater containing a significant amount of toxic aromatic amines causes ecotoxicity as up to 84% of PPD is left unused after hair dyeing which is an emerging environmental concern that has received little research and understanding [4-7]. In order to effectively manage the issue of related environmental risks as well as different health dangers, knowledge and understanding of environmental consequences are essential [8-10]. Abiotic and biotic variables, such as oxygen and microbes, often transform the parent contaminant into more toxic compounds [11]. Studies conducted by Aeby et al. [12], da França et al. [2], and Meyer and Fischer [13] and the National Center for Biotechnology Information (2019) have reported that approximately 3-5% of PPD autoxidizes in aqueous solution into combination of different toxicity polymers such as Bandrowski's base which is 10 times more toxic than PPD and also known to cause cancer (LD50 of PPD: 80 mg/kg; LD50 of Bandrowski's base: 5 mg/kg [12,13]. Therefore, there is a need for regulatory controls on PPD wastewater pollution from the hair dyeing industry to prevent potential environmental hazards.

The primary enzyme involved in the N-acetylation of HDs and their constituents, such as PPD (arylamine), is NAT1 [14,15]. The conversion of PPD into N-mono and N,N-diacetylated (MAPPD and DAPPD, respectively) metabolites was mediated by N-acetyltransferase-1 (NAT-1) located in the epidermis and N-acetyltransferase-2 (NAT-2) located in the liver and gut [16,17]. The conversion of aromatic amines to N-hydroxylamines by CyP450 oxidation is a crucial step in the activation of arylamines. This ultimately results in the creation of nitrenium ions by O-acetylation or sulfation, which can damage DNA and have a carcinogenic effect on the bladder [5].

HD-related side effects are becoming more common due to the growing trend of hair coloring. These effects can range from mild contact dermatitis that is limited to one body site (hand dermatitis) or widespread generalized dermatitis to serious, potentially fatal issues like contact urticaria/angioedema, rhinitis/bronchospasm/asthma, and renal toxicity [18,19]. According to International Agency for Research on Cancer (IARC), some in vitro and in vivo studies on exposed human subjects have shown that some HDs and various chemicals used in HDs are mutagenic or carcinogenic [5]. According to several studies, PPD causes blood, breast, and bladder cancer in humans and other mammalian species [6,20,21,22].

In addition, tattoos are a form of decorative body art created through the dermal injection of pigment. Tattoos that include aromatic amines (PPD) carry a number of health hazards, such as the potential for cutaneous viral infections that are limited to the tattoo ink; on the other hand, systemic diseases can be caused by viral pathogens obtained through injection [23].

There is great demand for HDs due to their ability to impart temporary or permanent change in hair color which satisfies the consumers' desire for beauty, fashion, and a look-younger image. PPD is a well-known cause of type 4 hypersensitivity reactions from topical contact and is a popular ingredient in temporary tattoos and HDs. Along with the increasing popularity of HD use, growing complaints about HD-induced hair loss have been a concern of dermatologists. H_2_O_2_, monoethanolamine, and PPD in HDs have been proposed as the main causative ingredients of hair dyeing-induced oxidative stress and cytotoxicity in human keratinocytes causing hair loss. Histological examinations of animal models have shown that oxidative stress is the reason for hair-dye-induced dermatitis and hair loss. H_2_O_2_ and monoethanolamine, in particular, were shown to synergistically induce oxidative stress and cytotoxicity in human keratinocytes.

In recent years, tattoos have gained enormous popularity among individuals worldwide. Tattoo artists inadvertently introduce a lot of unknown substances into the skin when they apply tattoo ink inside the skin with the use of small needles. These elements include heavy metals, polycyclic aromatic hydrocarbons, and primary aromatic amines, which are either created inside the skin through various processes like cleavage, metabolism, and photodecomposition, or accidentally entered with the ink. Group 2B substances include carbon black, cobalt sulfate, mercury, and other soluble cobalt salts that may cause cancer in people. On the other hand, Group 1 includes cadmium and its compounds (carcinogenic to humans). Various components of tattoo inks are harmful to human health or they become harmful after the way they are metabolized under the skin. Regulations and public awareness campaigns are needed globally to increase awareness about adverse health effects of tattooing. Zebrafish embryos are suitable animal models for studying how HDs affect embryonic growth. Studies have demonstrated that exposure to HDs induced morphological and physiological abnormalities in zebrafish embryos. Abnormal birth weight in humans (live birth weight <2500 g or >4000 g) reflects the poor health of the fetus and mother, which could contribute to the occurrence of obesity, malnutrition, hypertension, cardiovascular diseases, and cancer in the child in future. Hair dyeing and tattooing pose the risk of infantile abnormal birth weight that is elevated when mothers have irregular menstruation or have used HDs before pregnancy; the risk is increased if both factors exist.

Identification of correlates of poor knowledge, casual attitude, and wrong perceptions among HD users and those who prefer tattooing will help in reducing the prevalence of complications associated with their use. Published information on the PPD content of HDs and associated environmental and health risks are sporadic in both, animals and humans. Furthermore, there is a paucity of literature on the level of knowledge of ecotoxicity, health hazards, their PPD content, contact allergy, and practices of HDs and tattoos among college students. A comprehensive review of the literature only reveals a limited number of published reports regarding students’ knowledge of cutaneous viral lesions due to tattooing and the practice of getting tattoos that too not from KSA.

Moreover, information on knowledge of ecotoxicity, health risks, and contact allergy due to PPD content of HDs and tattooing among college students from Farasan Province is totally lacking.

About 40 kilometers offshore from Jazan is the stunning island of Farasan which is registered in UNESCO's Man and Biosphere Program. Farasan University College is affiliated to Jazan University. Despite its natural beauty, the scientific research outcomes are still less.

This study, therefore, was conducted with the aim to assess the knowledge of female college youth about ecotoxicity, health risks, and contact allergy due to PPD in HDs and the prevalence of hair dyeing and tattooing among these educated youth. 

## Materials and methods

This was a cross-sectional study conducted among undergraduate students at the Department of Nursing, English and Home Economics. The sampling procedure used was simple random sampling.

Research tool

A self-administered pre-tested questionnaire was used as a research tool for data collection from the study participants. Pre-testing of the questionnaire was done to evaluate the time needed to complete this assessment tool. Pilot survey results were not included in the final analysis.

The final self-administered questionnaire had 36 items, and answering them all took about eight minutes. The questionnaire covered items on demographic data which had six items including age, gender, qualification, study year, marital status and family’s educational background, participants’ knowledge, attitude, and opinion about HDs and allergies due to use of HDs if encountered.

In-depth information about preferred HD products and hair coloring frequency was also collected in the questionnaire. The questionnaire also requested detailed information regarding the brands of HDs preferred and the frequencies of hair dyeing. The questionnaire also requested details about performing an allergy test before using an HD and the motive behind hair dyeing. Safety-related questions concerned the use of HDs during pregnancy and lactation. The last section of the questionnaire was exclusively devoted to evaluation items on tattooing and its practices.

Sample size

In view of the current trend of hair coloring as a fashion statement among youth in Kingdom as per various previous studies and also according to the self-observation of the researchers, the required sample size was determined by Cochran’s formula, however, to compensate for a non-response rate, and 5% of the calculated samples were taken into account to get the final result of sample size which came to be 319.

Data collection procedure and ethical approval

A formal approval was taken from the College Ethical Committee (Farasan University College) before conducting the study (22/02/IRB/FUC.02). Enough information of research was provided to participants using informed consent via a consent form attached to the questionnaire. Consent was taken from all the participants and a free hand was given to the participants to participate in the study or refused to participate. Full confidentiality of the information was ensured. The rights of participants were protected as per the Helsinki Code of Ethics. The questionnaire was distributed among students of Farasan University College as Google Forms to their WhatsApp groups. The link was retained active for a month between January to February 2022.

Inclusion and exclusion criteria

Regular students enrolled with Jazan University during academic year 2021-2022, however, those who were not willing to participate were excluded from the study.

Statistical analyses

All statistical analyses were done using IBM SPSS Statistics for Windows, Version 17 (Released 2008; IBM Corp., Armonk, New York, United States). Descriptive statistics were used to report numerical variables as mean (SD) and categorical variables as frequencies (n) and percentages (%). To investigate the relationship between the frequency of HD use and demographic factors, the chi-square test for trend was employed. A score of one was given to each correct response; a score of zero was given to any wrong or possibly incorrect response. The means for each domain and the sum of the scores were then determined. A score above the mean was interpreted as indicative of good knowledge, and a score below the mean as poor knowledge. The reliability of the questionnaire was assessed using Cronbach's Alpha, which yielded a result of 0.875. A significance level of p<0.05 was used. The information was entered into a password-protected personal computer to guarantee secrecy. The information was entered onto a password-protected personal computer to guarantee confidentiality.

## Results

The demographic characteristics of the study participant are given in Table 1. Analysis of responses (Table 2) revealed that 61% (n=195) of respondents agreed that HDs and tattoos are unsafe. However, about their carcinogenic potential, only 24% (n=77) of respondents were agreed. Sixty-seven percent (n=214) of the participants were not aware of the risks of HDs during pregnancy and lactation (Table 2) and a small portion of the participants dyed their hair during their pregnancy (9.3%, n=30). Seventy-eight percent (n=249) of study participants had no idea of the PPD content in hair dyes and tattoos, and only 38% agreed with their toxic effects (Table 2).

**Table 1:** Demographic information of study participants (n = 319). N: Number of participants; Mean±SD (N=319)

Parameters	Frequency (N)	Percentage (%)
Mean age (21.8±3.2 years)		
≤20 year	216	82.9
>20 year	44	17.1
Gender		
Female	319	100%
Male	-	-
Marital status		
Married	57	18
Unmarried	262	87
Residence		
Farasan	97	56.6
Jazan	80	36.8
Other cities	142	6.6
Living with parents		
Yes	161	50.5
No	158	49.5
Study year		
I^st^ year	32	09.90
II^nd^ year	52	16.20
III^rd^ year	63	19.8
IV^th^ year	172	54.0

**Table 2:** Knowledge of hair dyes and tattoos, frequencies of HD practices, impact of hair dyeing on hair quality and body, and reasons for hair dyeing (n = 319). *: Response as Yes, **: Response as No, N: Number of participants; Mean±SD (N=319), p<0.05

Knowledge of Hair dyes and Tattoos among the study participants (n = 319).		
Variables	Frequency (N)/(%)	^#^p-value
Do you know about PPD content in HDs and Tattoos?	249/(78.40%)	^0.03^
Do you know about the negative environmental impact of HD?	121/(38%)*	^0.01^
Do you know the toxic effects of PPD contents in HDs & Tattoos?	121/(38%)*	^0.05^
Have you practiced HD during pregnancy?	30/(9.3%)*	^0.05^
Are the hair dyes and tattoos unsafe?	195/(61%)*	0.03
Can hair dyes and tattoos cause cancer?	79/(24.8%)**	0.02
Can hair dyes be used during pregnancy and lactation?	214/(67%)*	0.01
Do you perform allergy/patch test before using hair dye or tattoos?	19/(6%)**	0.02
Do you read the instruction manual before using hair dye?	93/(29%)*	0.04
Have you ever practiced tattoos?	12 (3.90%)*	
Have you ever experienced any adverse effect with hair dye and tattoos?	36/(11.3%)*	0.03
If yes, did you consult a medical practitioner for the same?	13/(4%)*	0.02
Reasons for not consulting a doctor		
Side effects were not too bad	168/(52.8%)*	0.03
Thought that these are the part of hair coloring and nothing serious	35/(11.1%)*	0.04
Took self-medication	09/(2.77%)*	0.01
Discontinued hair dye after side effects	29/(9.01%)*	0.03
Switched the brands	62/(19.4%)*	0.02
Frequencies and % of HD practices among study participants (n = 319)		
Monthly	57/(17.86%)*	0.01
< a month	78/(24.45%)*	0.02
Every fortnightly	39/(12.22%)*	0.03
Once in year	13/(4.06%)*	0.01
Never practiced	132/(41.4%)*	0.04
Impact of Hair dyeing on hair quality and body (n = 319)		
Hair loss	129/(40.5%)*	0.05
Dry hair	69/(21.6%)*	0.01
Dandruff	51/(16.0%)*	0.03
No side effect	69/(21.6%)*	0.12
Health		
Itching	149/(46.8%)*	0.01
Bruising	09/(2.7%)*	0.02
Red rashes	26/(8.1%)*	0.04
Puffy face	26/(8.1%)*	0.02
Eye infection	14/(4.5%)*	0.06
Increased heart rates	09/(2.7%)*	0.13
Tiredness	09/(2.7%)*	0.01
Nothing	104/(32.5%)*	0.07
Reasons for hair dyeing		
As a fashion statement	279/(87.5%)*	0.03
To cover grey hair	13/(04.0%)*	0.25
Younger look	06/(02.0%)*	0.07
Recommended by friends	22/(07.0%)*	0.03
Significant at p-value < 0.05; ^*^Significant at p-value < 0.05 based on the Chi-square test. Mean±SD (N=319). *=response as Yes, **=response as No		

Of those participants who responded to applying hair color, 24% were using it with a gap of less than a month (Table 2). In response to the question of whether they conducted an allergy test before using a HD product, just 6% (n = 19) of the participants claimed they had done so, and only 29% (n = 93) said that they had read the application instructions beforehand. When asked about the negative impacts of using HD, 12% of respondents (n=38) mentioned negative environmental and health impacts. Itching was the most frequent side effect (n=149, 46.8%), followed by hair loss, dry hair and dandruff (Table 2). Of the 36 (11%) participants who reported experiencing side symptoms, only 4% (n=13) sought medical attention from a professional.

When asked why they had not seen a doctor, 52.77% (n=168) said that the side effects were not too bad. The other respondents (11.1%, n=35) thought the side effects were part of hair coloring, and the remaining respondents (2.77%, n=9) took self-medication to deal with the same problem. Merely 29 participants (9.01%) discontinued their use of hair dyes, 62 (19.44%) switched brands, and the remaining participants continued to use the same hair coloring agent despite the negative consequences.

A total of 161 (50.5%) participants responded that during hair dyeing, they dyed the global area of the hair. This pattern is suggestive of coloring hair just for a fun or fashion and style (Table 2). However, those who dyed hair at an interval of a fortnight were doing only root touch up (n=22, 7%).

Table 3 illustrates the correlation between study years in the program, age, marital status and level of knowledge of study participants. A very strong correlation was found between hair dye practice, study year, and age, whereas a weak correlation was noted between marital status and hair dyeing and tattooing practices.

**Table 3:** Correlation between hair dyeing and tattooing practices and the variables such as study year, age, and marital status.

Variables	Correlation coefficient	^#^p-value
First year	0.821	0.02
Mid-year	0.898	0.04
Final year	0.899	0.05
Age	0.767	0.05
Marital status	0.018	0.36
^#^Significant at p-value < 0.05 based on the Chi-square test. Mean±SD (N=319).		

## Discussion

Recently a very significant study conducted by Dhafiri et al. at King Faisal University, KSA has emphasized educational awareness regarding adverse health impacts of hair dyeing [24]. Untreated synthetic dyes released into aquatic environments reduce the light available for photosynthesis by primary producers, with consequential impacts on the whole food chain. In addition, dyes are also directly harmful to plants, animals, and humans, with human health implications including increasing allergy and cancer risk. The recognition that human skin is not an impermeable barrier for some topically applied substances initiated the investigation of percutaneous absorption/penetration of HDs and their ingredients. The concentration of PPD in HD formulation varies from 70 to 90% in stone HD and 2-10% in branded dyes [25]. Exact PPD concentrations in formulations are not known as most HD solutions are proprietary. PPD is an aromatic amine and is a common component of HDs and tattoos [26,27] which can cause skin contact allergy and asthma with impaired pulmonary function. PPD is also a common allergen and is commonly suspected to be the cause of HD contact allergy [28]. The existence of high levels of unreacted PPD increases the likelihood of allergic events and elevates the risk of PPD-related chemicals [29]. PPD penetrates deep into hair due to its strong protein-binding ability. Given that the human scalp and skin are not always resistant to the inherent components of HDs, such as couplers (resorcinol, m-hydroxyphenols) and primary intermediates (p-phenylenediamines, p-aminophenols), hair colors have the potential to cause detrimental to the human body. Acute, reproductive, and genetic toxicity are among the conditions of carcinogenicity, mutagenicity, and human systemic risk reported in vitro studies. The chemical reaction in hair with PPD and hydrogen peroxide leads to the formation of a potentially carcinogenic and completely mutagenic reaction product [30].

The allergic reactions due to PPD toxicities in HDs and tattoos may cause swelling, burning, and itching sensation spreading across the skin. Serious reactions to PPD can result in life-threatening reactions such as urticaria, anaphylaxis, rhabdomyolysis, laryngeal edema, severe metabolic acidosis, and acute renal failure [31,32]. Another concern is the site of application of HDs, where the major target of exposure is the skin epidermal layer, which is formed mainly by keratinocytes. These cells actively respond to allergens and ROS by the induction of inflammatory cytokines and by direct stimulation of the immune system [33]. PPD is also cytotoxic to dendritic cells [34] and human neutrophils [35]. However, results of the present study indicate that only 36% of study participants performed allergy tests before dyeing their hair. Regarding tattooing practices, cutaneous viral infections at the site of tattoos have been reported in previous studies [24,36,37,38,39,40,41] In addition, systemic viral infections may develop in individuals who acquire a tattoo [23,41]. Chye et al. have also demonstrated that ROS production induced by PPD causes cell cycle arrest in the G1 phase in human liver cells [42]. Also, Picardo et al. hypothesized that oxidative stress may play a role in the development of allergy, which was attributed to the exposure of cells to autoxidation products of PPD [43,44].

According to research by Alghamdi and Moussa, 82.6% of the participating females in the survey had dyed their hair at some time in their lives [45]. The study also concluded that the usage of HDs is quite common among Saudi Arabian females. Of the participants, 11.6% began coloring their hair when they were 15 years old or younger. Hair coloring was first done on children as early as seven years old. In a similar vein, Sosted et al. revealed that 74.9% of females used HD in a previous survey conducted in Denmark. In the Danish trial, HD was started as early as the child's first birthday [46].

In European, North American, and Asian general populations, 1 to 6% of all patients with dermatitis have been reported to be sensitized to PPD; these percentages rise to 38% to 97% in patients with HD dermatitis [25]. Another study reported a median prevalence of PPD sensitization in Asia of 4.3% [27]. These rates seem to be related to the increased demand for hair coloring and the trend for individuals to begin using HDs for cosmetic purposes at a relatively young age.

In addition, PPD can leave permanent scars sensitivity to chemicals. To help prevent or limit allergic reactions, the majority of these products recommend that users conduct an allergy/patch test before using the product. Most participants in this study did not do an allergy test before using any dye. This approach raises concerns because 8% of HD users experience post-dye allergy issues. Another concerning issue is that many of the participants did not use HDs as directed, which is concerning because improper or excessive usage of HDs might harm hair. In the present study adverse reactions on hair quality as dry hair were noted in 40.5% of the study participants. The spectrum consisted of rashes, burning, dry hair, hair fall, dandruff, puffy face, eyes infection and ears with itching being the commonest. In a study by Zaid et al., 20.4% of the women reported allergic reactions with itching and redness of the scalp being the commonest and the findings of this study are in line with various other studies done on HDs in the past [47].

The majority of respondents (77.4%) expressed dissatisfaction with their original hair color; in Saudi Arabia, black and dark brown are the most common natural hair color. Some individuals (22.6%) expressed dissatisfaction with their natural hair color. These results show that consumers continue to use HDs despite their lack of confidence in their safety and sometimes dissatisfaction with the results. None of the participants experienced an immediate or severe adverse reaction; however, 46.8% of participants reported that after 24 hours of the use of HDs, they experienced itching and dryness of hair (Table 2). A side effect of dye on the body of hair like dryness, hair fall, and change of texture was the important finding of this study. These results suggest that further research on the composition of hair and type of HD is necessary. It's also necessary to raise awareness among younger generations about the need to refrain from undue and frequent application of hair coloring.

Limitations

The main limitation of the study was the small sample size; a cross-sectional study sample should be large to get the accurate findings of results from participants of the study. Second, the self-report questions for young nurses were another limitation in this study; this mostly affected the study with biasness.

## Conclusions

Our study found a higher practice of hair dyeing with higher frequencies of applications among these educated youth as fashion statements, which reflects the higher exposure of the youth to PPD at this very young age. Practicing getting tattoos was also found, but with a very low prevalence.

There is an urgent need to create awareness regarding the ecotoxicity and adverse health impacts of HDs and tattoos to reduce any potential health impact on life quality.

## Appendices

**Table 4:** Sample questionnaire HD: Hair dye; PPD: p-phenylenediamine

Knowledge of hair dyes and tattoos among the study participants (n = 319).		
Variables	Frequency (N) (%)	^#^P-value
Do you know about PPD content in HDs and tattoos?		
Do you know about the negative environmental impact of HD?		
Do you know the toxic effects of PPD contents in HDs & tattoos?		
Have you practiced HD during pregnancy?		
Can hair dyes and tattoos cause cancer?		
Can hair dyes be used during pregnancy and lactation?		
Do you perform allergy/patch test before using hair dye or tattoos?		
Do you read the instruction manual before using hair dye?		
Which brand of HD you use frequently		
Have you ever experienced any adverse effect with hair dye and tattoos?		
If yes, did you consult a medical practitioner for the same?		
Reasons for not consulting a doctor		
Side effects were not too bad		
Thought that these are the part of hair coloring and nothing serious		
Took self-medication		
Discontinued hair dye after side effects		
Switched the brands		
Impact of hair dye on hair quality and associated health risk		
Dry hair		
Hair loss		
Dandruff		
None		
Itching		
Bruising		
Red rashes		
Puffy face		
Eye infection		
Increased heart rates		
Tiredness		
Nothing		
Have you ever practiced tattoos?		
Tattoo pigments are accumulated in the lymph nodes		
Different colored tattoos have different levels of risk		
Do you think bleeding or hematoma is caused due to tattoos		
Do you receive enough information before getting a tattoo done		
Frequencies and % of HD practices among study participants (n = 319)		
Monthly		
< a month		
Every fortnightly		
Once in year		
Never practiced		
Areas covered during Hair dyeing		
Entire hair		
Roots		
Partial		
Impact of Hair dyeing on hair quality and body (n = 319).		
Variables (Hair Quality)		
Hair loss		
Dry hair		
Dandruff		
No side effect		
Health		
Itching		
Bruising		
Red rashes		
Puffy face		
Eye infection		
Increased heart rates		
Tiredness		
Nothing		
Reasons of Practicing HD		
As fashion statement		
To cover grey hair		
Younger look		
Recommended by friends		
^#^Significant at P value < 0.05 based on Chi square test. Mean±SD (N=319). Response collected at 3 point scale as Yes, No & May be		
